# Exploring suicide among immigrant farmworkers in the United States: evidence from the National Violent Death Reporting System

**DOI:** 10.3389/fpubh.2026.1937891

**Published:** 2026-10-02

**Authors:** Sara A. Kohlbeck

**Affiliations:** Comprehensive Injury Center, Medical College of Wisconsin, Milwaukee, WI, United States

**Keywords:** farming, farmworkers, immigrants, mixed methods, research methodology, suicide

## Abstract

**Background:**

Agricultural workers face some of the highest suicide rates in the United States, yet limited research has examined suicide among immigrant and migrant farmworkers (IMFs), a population recognized as particularly vulnerable within occupational health surveillance priorities. This study examined circumstances contributing to suicide among IMFs and compared these to circumstances among U.S.-born farmworkers using National Violent Death Reporting System (NVDRS) data.

**Methods:**

This multiple methods study used NVDRS data from 2013 to 2023, restricted to decedents in agricultural worker occupations (SOC Minor Group 45–2000) who died by suicide. Decedents were classified as IMFs (foreign-born) or U.S.-born based on birthplace. Bivariate analyses examined associations between nativity and reported circumstances preceding suicide, followed by multivariable logistic regression adjusted for age, sex, race, ethnicity, marital status, and education. A single-author qualitative analysis of investigative narratives, using an iterative inductive coding process with peer debriefing as a rigor check, identified themes describing IMF suicide circumstances.

**Results:**

Of 1,162 decedents, 17% (*n* = 201) were foreign-born. IMFs were older, more likely male, more likely Hispanic, and had lower educational attainment than U.S.-born decedents. IMFs were less likely to die by firearm (22% vs. 57%) and more likely to die by hanging/strangulation/suffocation (64% vs. 30%). In adjusted analysis, Hispanic ethnicity (OR = 0.014), older age (OR = 0.961), and being married (OR = 0.366) were associated with lower odds, while firearm method (OR = 2.126) was associated with higher odds, of U.S.-born status. Investigative documentation completeness did not differ significantly by nativity. Qualitative analysis identified four themes: stigma and avoidance of mental health care; violent and conflictual intimate relationships; substance use and associated familial shame; and immigration-related vulnerability and compounded isolation.

**Conclusion:**

IMFs experience intersectional stressors—including immigration-related vulnerability, stigma, and isolation—that shape suicide risk in ways distinct from U.S.-born farmworkers, and that align with constructs of thwarted belongingness and perceived burdensomeness from the Interpersonal-Psychological Theory of Suicide. Predominant use of hanging/strangulation among IMFs highlights limitations of conventional lethal means restriction strategies for this population. Culturally responsive, community-based, and workplace interventions—including stigma reduction, social connection programming, and multilingual lethal means safety messaging—are needed to address suicide risk among this underrecognized and vulnerable occupational group.

## Introduction

Suicide remains a key leading reason for preventable death among the U. S. working-age population, and both mental health and substance use pose significant risks to the workforce in terms of disability, burnout and chronic disease burden (1–3) as well as proving costly to employers (4, 5). Agricultural workers have some of the highest suicide rates in the United States (6, 7) emphasizing the importance of tired efforts to address the behavioral health needs of this population. Despite significant research exploring suicide among farmers, less attention has been directed to suicide among immigrant and migrant farmworkers (IMFs) despite the fact that these workers have been recognized as a “vulnerable population” meriting special consideration within NIOSH’s National Occupational Research Agenda (NORA) for Agriculture, Forestry, and Fishing research (8). Such workers face many of the same stressors experienced by farmers and rural populations (9) while experiencing other structural stressors specific to their lived experience as IMFs.

There are many structural and institutional factors that fundamentally shape the context of farmworkers’ mental health risks. Farmworkers are among the least protected workers in the U.S. workforce, given the ways in which agriculture is exempted from most state and federal protections surrounding worker hours, age restrictions, health and safety enforcement, and workers compensation. While formal agricultural labor programs, such as H2A visas, exist for some seasonal crop agricultural workers, such programs have “not been a viable option or common human resources practice for the U.S. farming industry” (10). This means that such workers are often undocumented, rendering an already under-protected class of workers especially vulnerable.

While all undocumented immigrant workers face stressors related to their legal status, those working on farms face additional challenges. Farming has always been among the highest risk occupations, and the exigencies of modern farm operations perpetuate unsafe, unhealthy, and unsustainable working conditions. Low profit margins and farm consolidation (11) mean that most farms operate with a small number of employees—a U.S. average of 4.4 in 2023—covering 24/7 operations (10, 12, 13). As a result, working conditions for immigrant farm workers share many of the features of other non-standard work arrangements—including lack of control over scheduling, lack of benefits, and few legal protections (8).

Factors such as long hours, shift work and work-related stress are endemic to farm work. As noted in the NORA on Healthy Work Design and Well-Being report, “fast-paced work, high job demands, job insecurity, and low wages...may increase risks for poor mental health outcomes” (8). Thus, immigrant farmworkers often face stress-inducing job demands, physical strain and legal vulnerability. As immigrants often live on and around farms - and are frequently barred from obtaining a driver’s license – they are often linguistically, culturally and geographically isolated. These risk factors combine to make immigrant farm workers particularly susceptible to depression, anxiety, and substance use. These risks are compounded by the extremely high rates of occupational injuries in farm workers (twice the industry rate overall) (10), which can further leave workers struggling with chronic pain, disability, and lost wages—particularly given the industry’s general lack of paid sick leave and health insurance.

This mixed methods study proposes to close the gap in research focusing on suicide among IMFs by answering our key research question: What circumstances contribute to suicides among farmworkers not born in the US, and how do these circumstances compare to those contributing to suicides among US-born farmworkers? Findings from this study will be strategically disseminated to academic and industry audiences to guide the development, implementation, and evaluation of strategies to prevent suicide among IMFs, thereby contributing to the improvement of mental health and wellbeing for this population. The unique attributes of the National Violent Death Reporting System (NVDRS) will contribute to a broader, more complete understanding of suicides in IMFs by enabling the identification of risk factors unique to IMFs, and the understanding of contributing circumstances like workplace conditions, substance use, and social support systems, which will inform culturally appropriate prevention efforts and policies to prevent suicide in this vulnerable group.

## Methods

Dataset: This study uses data from the National Violent Death Reporting System (NVDRS). The NVDRS is a national violent death surveillance system, administered through the Centers for Disease Control and Prevention (CDC), that records information pertaining to suicides and circumstances preceding suicides, in addition to similar information for homicides, unintentional firearm injuries, violent deaths of undetermined intent, and legal intervention deaths (14). Data for this study were obtained via the NVDRS Restricted Access Database (RAD) through a data use agreement between the study investigator and the CDC.

Study Sample: For this study, we obtained data on all suicides occurring in the United States between 2013 and 2023. At the time the study was conducted, 2023 was the most recent data year available. To obtain our study sample, we first used the SOC2018_Major Group variable, and included those decedents who were working in farming, fishing, and forestry occupations at the time of their suicide (SOC Major Group = 45). The SOC2018_Major Group uses the U.S. Bureau of Labor Statistics Standard Occupational Classification System, wherein each occupation is placed within one of 23 major groups (15). We further defined our sample by using the SOC2018_MinorGroup variable and included those decedents who met the definition of Agricultural Workers (SOC Minor Group = 45–2000). This was done to exclude first-line supervisors of farm workers as well as those decedents working in fishing and forestry occupations.

We then used decedents’ birthplace to generate a binary born in the United States/not born in the United States to establish our study group and our comparison group. Any decedents whose place of birth was unknown or “not applicable” were excluded from the sample. Those not born in the United States comprise our IMF group for the purposes of this study.

Analysis: We calculated descriptive statistics to describe our sample and then conducted bivariate analyses examining the relationships between IMF status (birthplace) and the reported stressors preceding suicide using chi-square, Fisher’s Exact (for circumstances with cell sizes of 5 or less). Fully adjusted analyses were conducted using logistic regression and accounted for race, sex, and age, ethnicity, marital status, and education level. The outcome of interest was IMF status. Rather than selecting covariates for the adjusted model based on statistical significance in unadjusted analyses, we specified covariates *a priori* based on theoretical relevance and prior published literature. Specifically, variables were selected based on established associations with suicide as established in NVDRS-related literature (16, 17). Statistical analyses were conducted using STATANow/SE 18.5. *p*-values <0.05 are considered statistically significant.

An important consideration with NVDRS data is that the circumstantial data that is endorsed in the system includes circumstances that were shown to have contributed to the suicide. In addition, the non-endorsement of a circumstantial factor does not necessarily mean that a circumstance was not present in a decedent’s life. Rather, this indicates that there was not sufficient evidence available in the death investigation to support endorsing this circumstance (18, 19).

Qualitative analysis of investigative narratives was conducted by a single author using an iterative, inductive coding process. Coding proceeded in two rounds. In the first round, the author read all narratives in full and developed an initial set of codes grounded in the data, capturing recurring concepts, language, and circumstances described across cases. Codes generated in this round were then reviewed, compared, and refined, with collapsing overlapping or redundant codes and clarifying code definitions, to produce a consolidated coding structure. In the second round, this refined coding structure was applied systematically across the full set of narratives, with the coder returning to previously coded excerpts to ensure consistent application of code definitions as the coding scheme stabilized. Codes were then grouped and abstracted into broader candidate themes based on patterns of co-occurrence and conceptual relatedness across cases.

Because coding was conducted by a single author, intercoder reliability statistics were not applicable to this design. Instead, peer debriefing was used as a rigor check to support the credibility and trustworthiness of the analysis (after the initial coding structure was finalized and again during theme development). During debriefing sessions, a colleague with qualitative research experience who was not directly involved in coding reviewed the emerging codebook, representative coded excerpts, and candidate themes alongside the raw narrative data. This process allowed an independent reader to evaluate whether the assigned codes and themes were adequately grounded in and reflective of the underlying data, to probe the coder’s interpretations and analytic decisions, and to identify any alternative interpretations that may have been overlooked. Feedback from these sessions was used to refine code definitions, clarify theme boundaries, and strengthen the final thematic structure presented in the Results.

Qualitative analysis was conducted using MAXQDA 2022.

All study activities received a non-human subjects research determination from the Institutional Review Board at the Medical College of Wisconsin.

## Results

### Descriptive results

A total of 1,162 decedents were included in our study. Of those, 17% (*n* = 201) were born outside the United States. Males comprise a majority of the sample (93%, *n* = 1,084), and the mean age of decedents was 42.6 years old (SD = 18.4 years). Most decedents (*n* = 972, 84%) were White and 265 decedents (23%) were Hispanic. Overall, IMFs who died by suicide were older (mean = 49 years, *p* < 0.001), more likely to be male (96.5% vs. 92.6%, *p* = 0.04), more likely to be Hispanic (85% vs. 10%, *p* < 0.001), and less likely to have attained a higher level of education. In fact, half of IMFs had an educational level of 8th grade or less, whereas 52% of decedents born in the U.S. had a high school diploma. Interestingly, IMFs were less likely to use a firearm in their suicide than farmworkers born in the U.S. (22% vs. 57%, *p* < 0.001) but more likely to die by hanging, strangulation, or suffocation (64% vs. 30%, *p* < 0.001). Additionally, IMFs were more likely to be married or in a civil union than U.S.-born decedents (38% vs. 22%, *p* < 0.001). Full sample demographics are included in Table 1.

**Table 1:** Sample description.

Variable, *n* (%)	IMFs only, *n* (%)	Non-IMFs only, *n* (%)	*p*-value (IMF vs. non-IMF)
Sex	Male: 194 (97%); Female: 7 (3%)	Male: 890 (93%); Female: 71 (7%)	0.04
Race	White: 145 (72%); Black or African American: 12 (6%); American Indian/Alaska Native: 0 (0%); Asian: 7 (3%); Native Hawaiian/Pacific Islander: 3 (1%); Other/Unspecified: 31 (15%); Two or more races: 0 (0%); Unknown: 3 (1%)	White: 827 (86%); Black or African American: 39 (4%); American Indian/Alaska Native: 44 (5%); Asian: 2 (0.2%); Native Hawaiian/Pacific Islander: 0 (0%); Other/Unspecified: 16 (2%); Two or more races: 29 (3%); Unknown: 4 (0.4%)	<0.001*
Ethnicity	Not Hispanic: 28 (14%); White Hispanic: 127 (63%); Black Hispanic 11 (5.5); Unknown: 1 (0.5%)	Not Hispanic: 861 (90%); White Hispanic: 70 (7%); Black Hispanic: 0 (0%); Unknown: 3 (0.3%)	<0.001*
Marital status	Married/Civil Union: 77 (38%); Never Married: 88 (44%); Widowed: 15 (7%); Divorced: 18 (9%); Single, not otherwise specified: 1 (0.5%); Unknown: 2 (1%)	Married/Civil Union: 214 (22%); Never Married: 471 (49%); Widowed: 33 (3%); Divorced: 193 (20%); Single, not otherwise specified: 42 (4%); Unknown: 8 (0.8%)	<0.001*
Education level	8th grade or less: 99 (49%); 9th to 12th grade, no diploma: 36 (18%); High school graduate or GED: 44 (22%); Some college credit, no degree: 4 (2%); Associate’s degree: 1 (0.5%); Bachelor’s degree: 3 (1.5%); Master’s degree: 0 (0%); Professional degree: 0 (0%); Unknown: 13 (6%)	8th grade or less: 46 (5%); 9th to 12th grade, no diploma: 220 (23%); High school graduate or GED: 494 (52%); Some college credit, no degree: 95 (10%); Associate’s degree: 34 (4%); Bachelor’s degree: 36 (4%); Master’s degree: 3 (0.3%); Professional degree: 0 (0%); Unknown: 22 (2%)	<0.001*
Mechanism of injury	Firearm: 45 (22%); Sharp Instrument: 6 (3%); Blunt Instrument: 0 (0%); Poisoning: 12 (6%); Hanging, strangulation, suffocation: 129 (64%); Fall: 3 (1%); Drowning: 0 (0%); Fire or burns: 1 (0.5%); Motor vehicle: 4 (2%); Other transport vehicle: 0 (0%); Other: 0 (0%); Unknown: 1 (0.5%)	Firearm: 541 (57%); Sharp Instrument: 12 (1%); Blunt Instrument: 2 (0.2%); Poisoning: 61 (6%); Hanging, strangulation, suffocation: 286 (30%); Fall: 8 (1%); Drowning: 5 (0.5%); Fire or burns: 4 (0.4%); Motor vehicle: 8 (1%); Other transport vehicle: 11 (1%); Other: 3 (0.3%); Unknown: 1 (0.1%)	<0.001*
Age, MEAN (SD)	49 (21)	41 (18)	<0.001*
Mental health problem	61 (30%)	295 (31%)	0.9
Depressed mood	75 (37%)	238 (25%)	<0.001*
Current mental health treatment	17 (8%)	136 (14%)	0.03*
History of mental health treatment	34 (17%)	196 (20%)	0.3
Treatment non-adherence	2 (1%)	2 (2%)	0.3
Alcohol problem	41 (20%)	181 (19%)	0.6
Substance use problem	24 (12%)	170 (18%)	0.04*
Intimate partner problem	38 (19%)	230 (24%)	0.1
Family relationship problem	16 (8%)	68 (7%)	0.7
Other relationship problem	4 (2%)	24 (2%)	0.7
Abused as a child	0 (0%)	6 (0.6%)	0.3
Interpersonal violence perpetrator	7 (3%)	21 (2%)	0.3
Interpersonal violence victim	0 (0%)	1 (0.1%)	0.6
Argument	33 (16%)	163 (17%)	0.8
Precipitated by other crime	11 (5%)	37 (4%)	0.3
Suicide attempt history	35 (17%)	112 (12%)	0.03*
Suicide thought history	60 (30%)	260 (27%)	0.4
Suicide intent disclosed	36 (18%)	182 (19%)	0.7
Suicide note left	25 (12%)	185 (19%)	0.02*
Recent criminal legal problem	11 (5%)	100 (10%)	0.03*
Physical health problem	27 (13%)	119 (12%)	0.7
Job problem	5 (2%)	57 (6%)	0.05
Financial problem	7 (3%)	45 (5%)	0.4
Eviction or loss of home	1 (0.5%)	29 (3%)	0.04*
Recent suicide of friend or family member	0 (0%)	19 (2%)	0.06
Death of a friend or family member	10 (5%)	48 (5%)	1.0
Traumatic anniversary	1 (0.5%)	6 (0.6%)	0.8
Any crisis	43 (21%)	256 (27%)	0.1
Marijuana result	7 (6%)	90 (20%)	<0.001*
Cocaine result	14 (11%)	22 (5%)	0.02*
Opioid result	8 (7%)	57 (13%)	0.07
Blood alcohol content (mean, SD)	0.45 (204)	0.55 (225)	0.6

IMF = Immigrant farmworker. *Statistically significant at *p* < 0.05.

### Analytical results

Bivariate analyses demonstrated significant associations between IMF status and the circumstances of depressed mood (*X*^2^ = 12.3, *p* < 0.001), current mental health treatment (*X*^2^ = 4.7, *p* = 0.03), substance use problem (*X*^2^ = 4, *p* = 0.04), suicide attempt history (*X*^2^ = 5, *p* = 0.03), suicide note left (*X*^2^ = 5.2, *p* = 0.02), recent criminal legal problem (*X*^2^ = 4.7, *p* = 0.03), marijuana result (*X*^2^ = 18.4, *p* < 0.001), and cocaine result (*X*^2^ = 9.8, *p* = 0.02).

We conducted multivariable analysis that included variables based on theoretical relevance. The outcome of interest in this analysis was IMF status. Table 2 displays results from this analysis. Overall, several demographics and circumstances remained significant, including Hispanic ethnicity, age, marital status, and method of suicide (firearm).

**Table 2:** Multivariable analysis.

Predictor	Reference category	aOR	95% CI	*p*-value
Hispanic ethnicity (yes)	Non-Hispanic	0.014	0.008–0.024	<0.001
Age (years, continuous)	—	0.961	0.947–0.975	<0.001
Married (yes)	Not married	0.366	0.213–0.629	<0.001
Firearm as method (yes)	Other method (e.g., hanging/suffocation)	2.126	1.268–3.563	0.004
Job problem (yes)	No job problem	2.00	0.57–6.94	0.277
Legal problem (yes)	No legal problem	1.60	0.62–4.18	0.334
Depressed mood (yes)	No depressed mood	0.73	0.44–1.22	0.230
Intimate partner problem (yes)	No IPV problem	1.28	0.70–2.33	0.425
Mental health problem (yes)	No mental health problem	1.17	0.69–1.99	0.561
Financial problem (yes)	No financial problem	1.32	0.40–4.37	0.645
Physical health problem (yes)	No physical health problem	1.18	0.53–2.64	0.679
Alcohol problem (yes)	No alcohol problem	0.85	0.47–1.52	0.582
Suicide note (yes)	No suicide note	0.78	0.41–1.51	0.467
Prior suicide attempt (yes)	No prior attempt	0.97	0.48–1.94	0.922
Female	Male	1.06	0.37–3.08	0.909
Year (centered on 2018, continuous)	—	1.02	0.94–1.11	0.651

*Statistically significant at *p* < 0.05.

Race was excluded from the multivariable analysis as it’s nearly collinear with Hispanic ethnicity in this sample. Specifically, nearly all Hispanic decedents in this sample were of White race.

Additional *post-hoc* analyses were conducted to evaluate whether investigative documentation completeness differed by nativity, we compared rates of unknown/undetermined circumstances and narrative text availability between foreign-born and U.S.-born decedents. Overall, 16.2% of U.S.-born decedents (156/961) had circumstances classified as unknown or undetermined (CircumstancesKnown_c), compared with 10.9% of foreign-born decedents (22/201); this difference did not reach statistical significance (χ^2^ = 3.19, *p* = 0.074), though the direction was opposite to what would be expected if documentation were systematically less complete for foreign-born decedents. Free-text investigative narrative fields (law enforcement and coroner/medical examiner circumstance text) were sparsely populated overall, with text present in only 6.0% of foreign-born and 7.2% of U.S.-born cases (χ^2^ = 0.21, *p* = 0.65); narrative length among cases with any text also did not differ significantly by nativity (*p* = 0.47 for coroner/medical examiner text; *p* = 0.42 for law enforcement text). Therefore, it does not appear that investigative documentation is systematically less complete for IMF decedents in this cohort.

### Qualitative findings

Through our qualitative analysis, we uncovered five themes, which are defined along with emblematic narrative excerpts below:

- “Doctors are for old women.” – Stigma or avoidance of mental health care, even when that care was needed;
- Significant, often violent, relationship issues contributed to suicide among IMFs;
- Substance use was prevalent among the deceased, and the familial shame associated with this substance use was an additional stressor;
- Immigrants faced identity-based social isolation, which was exacerbated by language barriers and missing family far away;
- Legal issues that immediately preceded the suicides were complicated by fear of deportation or other complications related to immigration status.

Notably, narrative data were missing or not available in 24% of the cases (*n* = 49).

Theme 1: *“Doctors are for old women”: Stigma and Avoidance of Mental Health Care*. Stigma played a large role in avoidance of help-seeking for IMF suicide decedents, so in many cases, needed mental health services were not accessed. While stigma was not the only factor impeding access to services among IMFs, it was sometimes strong enough to prevent help-seeking even among those with a previous suicide attempt. In one instance, a 41-year-old male farmworker whose family suggested he visit a mental health provider after expressing suicidal ideation, but he resisted due to stigma:

[Witness] recalled that once the victim once tried to kill himself in Mexico with a gun…the victim asked [the witness] to have a smoke outside with him when he stated that he was tired and having a lot of bad feelings. She told him to talk to someone, but the victim stated, “Doctors are for old women.”

Just three days prior to this suicide, the decedent was seen carrying a gun in his belt and had expressed suicidal intent to the witness and others. While it is not certain that seeking help in this time of crisis would have prevented this suicide, it is possible that clinical intervention may have benefitted this decedent.

Another decedent, a 71-year-old Hispanic male, had also expressed feelings of sadness and suicidal ideation in the months prior to his suicide but similarly refused to seek help for his mental health challenges:

[Victim] had made other threats of suicide and had attempted suicide earlier in the year according to his family. Victim was never diagnosed with depression but according to his daughter, he would never go to “that doctor’. Victim’s daughter also mentioned that Victim had recently visited a heart doctor and received some sort of examination but would not share the results with his family.

In this case, “that doctor” was a mental health provider, and the decedent’s reticence to seek care in this manner, while at the same time receiving care from another provider for heart issues, is evidence of stigma around mental health care. The fact that this decedent was also not willing to share results of his heart examination with family could reflect perceived burdensomeness. It is also possible that the decedent was not screened for suicide, or did not disclose his suicide history, when seeking care for his heart issues.

In a third case, a 60-year-old male who died from firearm suicide had been living with undiagnosed mental health issues for many years, and had a stroke several years before his suicide that also affected his mental health. However, the decedent was not willing to seek care for these challenges:

The victim had had a stroke 14 years prior and had never been the same, according to his oldest daughter. He also had undiagnosed mental health issues and believed that Witness 1 and his wife had been setting off bombs after hearing fireworks in the area a few weeks prior. The victim had refused to seek medical help for his mental illness. He appeared to be paranoid and delusional, and acted like he was hallucinating.

The magnitude of the mental health issues this decedent was experiencing prior to his suicide may be difficult to manage without professional support, and his refusal to seek care in spite of these difficulties may be representative of stigma among IMF suicide decedents.

Theme 2: *Violent and Conflictual Intimate Relationships Relationship* issues are a commonly stated factor contributing to suicide in the general population, and this is true among IMFs as well. However, in this sample, these relationship issues often resulted in or were born out of violence, adding a layer of seriousness to these experiences. For example, one 24-year-old male IMF died by suicide amidst relationship issues including a domestic disturbance:

Per police, victim was known to police due to an unspecified domestic disturbance with his ex-girlfriend 9 days prior to pronounced death during which victim was warned by police not to return to the ex-girlfriend’s town. Per police and victim’s coworker, victim had been sad about his breakup with his ex-girlfriend…Per police, victim’s social media messages showed that victim was upset about his breakup.

It was also noted that this decedent did not have any family living in the United States, potentially contributing to the impact of this relationship loss on the decedent’s life.

Family discord sometimes leads to violence, and this experience preceded suicide among one 22-year-old Hispanic male IMF. In this case, the decedent was arguing with his father and the argument escalated into gun violence:

Witnesses to the event stated the Victim had a drug problem and when his father told him to clean up a mess in the yard, they got into an argument and the Victim shot his father. The brother stated the victim doesn’t work, stays home and doesn’t do much around the house. The victim has been seeing a doctor or therapist for schizophrenia and was taking medication but stopped taking the medicine. The victim’s father always pushed the victim to be working. The victim’s mother has been suffering from cancer and is unable to work. The brother stated that the victim and their father bickered a lot because the victim would not work.

This suicide occurred in the context of multiple intersecting family stressors, including substance use, the mother’s cancer, mental health issues, and potential financial issues due to the decedent and his mother being unable to work. The violent nature of this final interaction between the decedent and his father, which was witnessed by the brother and mother of the decedent, created a crisis in which the decedent may have not seen any way out other than to take his own life.

Comorbid substance misuse and mental health challenges were also factors contributing to the suicide of a 28-year-old male IMF decedent who was a frequent perpetrator of intimate partner violence:

Victim is reported to be a heavy drinker of alcohol and suffers from depression. Victim has a long history of physical abuse of his wife. Victim had beaten his wife before his death and asked for her forgiveness. Victim’s wife states she refused to forgive him at which time victim went into the bathroom and secured the door. Wife later went to check on victim and found him [dead].

Research has examined the relationship between intimate partner violence perpetration and suicide (20) and some have suggested that suicide in this context may represent the ultimate form of coercive control that an intimate partner violence perpetrator exerts over his or her victim (21).Indeed, in this case, the decedent took his own life after his wife refused to forgive him, potentially as a way to try to force this forgiveness through his own death.

Theme 3: Substance Use and Associated Familial Shame Although our statistical analysis did not reveal an association between IMF status and alcohol problems or substance use problems among our suicide decedents, these issues were common among IMFs. This substance use was often the cause of guilt and shame, particularly when family members or loved ones of the decedent were aware of the extent of the problems Sometimes, this substance use was the cause of discord with family members:

30-year-old Hispanic male was found deceased…victim’s wife reported that victim had been drinking heavily every night in the month prior to this incident. Victim had gone to the bar this evening and upon returning home, Victim’s wife argued with Victim about his drinking and stated she was leaving to stay with other family. Victim had told his wife that she “should not be the one leaving, it should be me” right before going outside.

The prospect of losing his wife due to his heavy alcohol use as well as the shame associated with this stress appears to be a main contributing factor to suicide for this IMF decedent.

In another case, family members of a 57-year-old IMF decedent had argued with the decedent about this alcohol use immediately prior to his suicide:

According to Victim’s wife she and Victim had argued about his drinking, and she went outside to cool off. Another family member advised that he also spoke with Victim about his drinking prior to the incident, but Victim disagreed that he had a problem.

This confrontation with multiple family members, particularly when the decedent did not agree that his drinking was a problem, resulted in anger and may have contributed to a sense of shame in the decedent. In this case, the decedent was suspected of using alcohol in the hours prior to the suicide, which may have also affected his decision to take his own life.

Religion was also cited as a reason for shame related to substance use among IMF suicide decedents. One 22-year-old male IMF died by suicide after being confronted by his parents about how his consuming alcohol in the home was considered disrespectful for religious reasons:

He was last known alive the day prior when his parents confronted Victim about his alcohol consumption. Victim’s father reportedly spoke with Victim about the religious reasons why consuming alcohol in the house was an “indication that he does not show any respect.” A suicide note was found at the scene that read, “I’m sorry.” An open can of beer was also observed. Victim’s toxicology was positive for alcohol.

The decedent’s suicide note may be evidence of the shame he felt about his alcohol use and the fact that the decedent had alcohol in his system when he died may also reflect his reliance on alcohol to help him cope. In this situation, the decedent may have been experiencing not only perceived burdensomeness and shame, but also hopelessness in his ability to manage this situation in a way that would be considered respectful to his family and his religion.

Theme 4: Immigration-Related Vulnerability and Compounded Isolation IMFs often come to the United States to work without families are loved ones. This experience can be inherently isolating, and when combined with other stressors, this isolation may become incredibly difficult to bear. In addition, in many instances, IMFs are not United States citizens, and many IMFs are undocumented. In the context of our political climate over the past several years, being an undocumented individual in the United States sometimes involves a significant amount of stress related to the potential for deportation or other negative outcomes related to immigration status (e.g., being separated from loved ones). IMFs may not have access to the resources needed to obtain documentation to remain in the United States, and this is made more difficult by the often-transient nature of the work in which IMFs engage. When IMFs are faced, then, with the prospect of engagement with formal systems, including the legal system, the perceived or actual threat of deportation or other actions may become more prominent in the lives of IMFs. This stress was a contributing factor to suicide among a 60-year-old male IMF:

The victim had been in a depressed mood due to a recent DWI. He was afraid of being deported. The victim’s son was also in jail awaiting deportation for a DWI.

In this case, the threat of deportation was very real, given that the decedent’s own son was facing this reality at the time of the decedent’s suicide.

A 25-year-old IMF from Africa who died by suicide was dealing with multiple stressors, including the loss of a relationship, and these stressors seem to have been amplified by isolation from family and loved ones:

The victim lived alone, worked for a farmer, and had no family in the USA as he was from [Africa]. The evening prior, the victim had been at the bar drinking alcohol and arguing with his girlfriend/ex-girlfriend. The victim had been dealing with depression.

Living alone likely also contributed to the sense of isolation experienced by this decedent.

Another decedent, a 47-year-old Hispanic male IMF, reported having trouble communicating with his family, still living in his country of origin, which caused significant distress in the days preceding his suicide:

Law enforcement stated the suicide note indicated the time of Victim’s suicidal act, apologies to his friends and family and expression of his unhappiness with life. Victim’s wife told LE that Victim had been depressed and withdrawn lately because he hadn’t been able to communicate with his family that lived out of the country.

Geographical separation from loved ones can be very difficult, and when communication is not possible, this experience can be particularly stressful. Although this decedent was able to live with his wife in the United States, the separation from other loved ones led to depression and withdrawal, further compounding the isolation he felt.

Similarly, when conflict with loved ones does occur, geographical separation can make reconciliation more difficult. A 35-year-old IMF took his own life after arguing over the phone with his wife, who lived back in his country of origin:

Friends and co-workers reported that Victim had been talking with his wife in another country, and that Victim and his wife were involved in a verbal dispute on the night before. Victim had told them that without his wife he had nothing to live for.

Without other close loved ones to rely on for connection either in the United States or back in their country of origin, IMFs may feel particularly isolated and vulnerable, leaving them feeling like they are without a reason to live.

## Discussion

The purpose of this mixed methods study was to better understand factors contributing to suicide among immigrant farmworkers in the United States, and to explore how these factors may differ from those contributing to suicide among US-born farmworkers. To our knowledge, this is the first study to leverage NVDRS data to explore this phenomenon, and the first study to qualitatively examine investigation reports from suicides among immigrant farmworkers. We found significant differences in the demographic characteristics of IMF suicide decedents versus US-born farmworkers. Specifically, IMFs who died by suicide tended to be older, male, and have less educational attainment than US-born farmworker suicide decedents. Importantly, IMFs tended to die by suicide via hanging/strangulation whereas US-born farmworkers most often used a firearm in their fatal suicide attempt.

There is extensive research literature focusing on suicide in farming from a broad sense in the United States. These studies have identified several factors associated with this population, including burnout and hopelessness (22), experiences of depression (23), social factors including stigma and norms related to masculinity (24, 25) and individualism (26), physical health issues (27), alcohol use (26), access to lethal means (28, 29), and mental health challenges (30, 31). However, examining suicide among farmers and the farming community from the perspective of the farming community as an aggregate likely obscures important nuances in circumstances contributing to suicide among subpopulations of the farming community. Immigrant farmworkers comprise an important population of this broader community, and our study demonstrates key differences in factors contributing to suicide in this group. Specifically, our qualitative themes point to intersectional stressors that influenced and contributed to suicidal behavior among IMFs. Being an immigrant to the United States, particularly if that immigrant is undocumented, introduces additional vulnerabilities that may increase susceptibility to suicidal behavior. Importantly, anti-immigrant stigma and racism has been shown to exacerbate health disparities, and when considering suicide among IMFs, this stigma is a distinctive factor when compared with other populations within the farming community (32). This is a structural factor relevant to any suicide prevention efforts in this space.

When considering theoretical frameworks around suicide and suicidal behavior, our findings align with the Interpersonal-Psychological Theory of Suicide (6, 33). Specifically, the vulnerabilities associated with being an immigrant farmworker in the United States may contribute to a sense of thwarted belongingness and isolation. This isolation, loneliness, and sense of longing for loved ones living far away was a main contributor to suicide among several IMFs in this sample. Similarly, the lived experience of being an immigrant to the United States, particularly if one is highly dependent on others for their survival and livelihood, may contribute to perceived burdensomeness. Indeed, this phenomenon has been studied among Hispanic adults in the United States. One study found a strong correlation between perceived burdensomeness and suicide risk among Hispanic adults, and that ethnic identity mediated the relationship between perceived burdensomeness and suicide in this group (34). Given these findings, emphasizing and promoting a sense of ethnic identity among IMFs as part of suicide prevention initiatives may not only mitigate a sense of perceived burdensomeness but may also contribute to a sense of community and belonging, thereby addressing thwarted belongingness. However, in the current sociopolitical context of the United States, emphasizing ethnic identity outside the dominant paradigm in any public manner could be perceived as risky or dangerous to IMFs, and care should be taken in considering this approach.

The third component of the Interpersonal-Psychological Theory of Suicide is capability for suicide, which is achieved through repeated exposure to painful or provocative experiences (9, 35). Indeed, the act of immigration itself may be considered such an experience, particularly if one leaves one’s country suddenly out of necessity and/or without the support or presence of loved ones. However, other experiences aligned with capability for suicide were present in our sample. Substance use has been linked with fearlessness and pain tolerance, two important constructs in capability for suicide, and substance use was highly prevalent among our IMF suicide decedents (36). However, an additional experience of shame was sometimes linked with substance use in our sample. This sense of shame related to substance use often originated with family and sometimes resulted in loss of relationships, compounding any existing sense of thwarted belongingness. Substance use is a highly stigmatized experience (37) and the intersection of vulnerability associated with IMF status and the stigma associated with substance use is an important factor to consider in the context of suicide in this group.

Another prevalent experience among IMFs who died by suicide was interpersonal violence perpetration. Fearlessness about death is also positively associated with physical violence perpetration in men in particular (36), providing important context around the link between violence perpetration and suicide among IMFs in our sample. Another contextual factor here that is important to mention are the cultural norms related to interpersonal and intimate partner violence and hypermasculinity (38). As nearly 90% of IMFs in our sample were of Hispanic ethnicity, *machismo*, and its associated hypermasculinity may have played a factor in violence perpetration among suicide decedents in our sample, as *machismo,* and its associated relationship challenges, have been linked with suicide among decedents in Mexico (39). It is important to note here that intimate partner violence is also a stigmatized experience (40) and it is possible that the tension between *machismo* and the stigma of violence perpetration and/or victimization likely resulted in distress for IMF suicide decedents.

Our theme focusing on stigma around and/or avoidance of seeking help for mental health challenges is also likely in part associated with cultural norms around masculinity. Again, mental health stigma does not affect help-seeking among IMFs only, and studies have demonstrated how stigma plays a role in help-seeking across cultures, particularly among men (41, 42). In fact, researchers have examined the role of stigma in accessing mental health care among male farmers in the United States and in other countries (25, 26, 43). Similarly, endorsement of traditional norms around *machismo* are indeed associated with decreased willingness to seek help for mental health concerns among Hispanic men (44), and this likely played a role in our sample as well. However, whereas the mental health stigma or avoidance of mental health care support among farmers in the United States are at least in part rooted in a sense of rugged individualism (26, 45), the *machismo*-based stigma and avoidance of mental health care may be more rooted in traditional conceptions of gender, in which men are expected to be stronger than and dominant over women (46). This is illustratively supported in one narrative excerpt, in which a decedent noted that “doctors are for old women,” highlighting the gendered belief that help-seeking is not only just for women, but specifically old women, ostensibly those who may be seen as weak and feeble. However, little is known about how the concept of *marianismo* and similar gender-based constructs align with the Interpersonal-Psychological Theory of Suicide, and additional research in this space is warranted.

Most IMF decedents in our sample died via hanging, strangulation, or suffocation. This is a departure from suicide trends observed nationally, wherein firearms are the primary mechanism of injury involved, specifically among male suicide decedents (47). However, firearm suicide is highly predominant primarily in the United States, and as the IMFs in our sample were all born elsewhere, the distribution of mechanism of injury in this study align with what see in many countries around the world. In fact, proportions of suicide by hanging range in Latin American countries and are as high as 77% among males in Chile (48). Additionally, a post-COVID analysis of suicide in Mexico found that hanging is the most prevalent suicide method in that country, comprising approximately 73% of all suicides (49). This is highly relevant from a suicide prevention perspective. Limiting access to lethal means, and specifically firearms and poisonous substances, remains a prominent focus of suicide prevention activities in the United States. The same lethal means access strategies that are effective in reducing firearm suicide will likely not be as effective for hanging suicides, requiring a consideration of more upstream approaches that focus on preventing individuals from reaching a suicide crisis in the first place.

However, unlike limiting access to firearms or pesticides, hanging/strangulation lacks a discrete means that is amendable to regulation or secure storage practice. The objects used in hanging are integrated into everyday physical environments, including farming environments and cannot be completely eliminated or removed. This reality creates an important gap in lethal means security strategies for hanging specifically and reinforces calls in the research literature for other primary, secondary, and tertiary prevention strategies (e.g., crisis intervention, regular screening for suicide, emergency response to non-fatal attempts) rather than simply relying on restricting access to lethal means.

Our finding that Hispanic ethnicity was the dominant predictor of foreign-born status among farming, fishing, and forestry suicide decedents (OR = 0.014, 95% CI: 0.008–0.024) is consistent with national data on the demographic composition of the U.S. agricultural workforce. According to the National Agricultural Workers Survey (NAWS), the most comprehensive federal source on hired crop labor, approximately two-thirds of U.S. crop workers are noncitizen immigrants, and nearly half (47%) lack work authorization altogether. Earlier NAWS cycles similarly estimate that only about one-third of crop workers are U.S. citizens, with the remainder holding legal permanent resident, temporary visa (e.g., H-2A), or undocumented status. Given that noncitizen and undocumented status is disproportionately concentrated among Hispanic, foreign-born agricultural workers, the strong association we observed between Hispanic ethnicity and foreign-born status likely reflects this underlying occupational demographic structure rather than a direct effect of ethnicity itself. This pattern underscores that nativity-related disparities in our sample are best interpreted as markers of broader structural and labor-market conditions, including immigration status, work authorization, and associated social and economic vulnerability, rather than as independent risk factors for suicide. Notably, NAWS data capture only hired crop labor and therefore may not fully represent those included in our sample, and legal status in NAWS is self-reported under conditions designed to encourage disclosure.

Our findings present a number of public health and occupational health implications for suicide prevention among IMFs. It has been well-established that social connection and a sense of belonging are protective not only against suicide and suicidal behavior, but also have the potential to reduce other risky health behaviors, including alcohol and other substance use. Interventions that increase social connection among IMFs may be effective in mitigating thwarted belongingness in this group, and connections with those sharing an ethnic identity with IMFs may prove especially helpful. Programming that also incorporates healthy coping and stigma reduction around help-seeking is warranted. An example of this type of program, *Bienvenido*, developed in the State of Indiana, focuses on a strengths-based approach to mental health promotion specifically for Hispanic immigrants. Bringing IMFs together to enhance connection while learning skills to promote and protect mental health through a culturally relevant curriculum could address several contributing factors to suicide in this group. However, *Bienvenido* has not been evaluated as a suicide prevention strategy, so future work in this space is needed.

As mentioned, many suicides among IMFs occurred via hanging, which is difficult to address with typical lethal means access strategies (e.g., gun locks, secure storage). At the same time, roughly one in four IMF suicides did occur using a firearm. It is worth noting here that undocumented immigrants, including individuals in the United States on a nonimmigrant visa, are prohibited from possessing a firearm under 18 U.S.C. 922(g)(5), and in the cases of IMF firearm suicide, this policy was not a sufficient deterrent to obtaining and using a firearm in a fatal suicide attempt. Therefore, messaging around lethal means storage and safety must be tailored to meet the needs of IMFs. This includes not only messaging in languages other than English but also messaging that emphasizes the safety considerations of firearms in the home and in the workplace. Firearms are often used as a “tool of the trade” in farming spaces, making this highly lethal method readily accessible (26). Therefore, lethal means safety strategies should also involve farm owners and managers, educating them about the implications of easily accessible firearms in the context of suicide prevention among their workforces.

This study has a number of limitations that are important to consider. One limitation is related to the missingness of narrative information, which not only limits the availability of qualitative information, but also contributes to underreporting of circumstantial variables within the NVDRS. As NVDRS circumstances are only included if there is sufficient evidence within a case report to support it, a lack of investigation information among IMFs specifically introduces systematic bias into this study. Other studies have documented how decedent characteristics (and specifically race) are significantly related to the presence and length of narrative text in the NVDRS, with White, non-Hispanic suicide cases having narratives present most often. This is highly problematic, as the narrative information contained in the NVDRS is often rich and is instrumental in better understanding contributors to suicide in the United States, thereby informing suicide prevention activities. A lack of narrative information, particularly when the absence of this information disproportionately affects minoritized groups, may exacerbate existing disparities in suicide or lead to ineffective or poorly planned prevention efforts. NVDRS narratives originate from suicide investigations, and much of the information generated in these investigations comes from family members and loved ones of the deceased. In the case of IMF suicide, it is reasonable to suggest that language barriers present a real problem in obtaining rich contextual information on contributors to suicide in this group. Including language interpreters as part of a team that responds to suicide among non-English-speaking decedents may prove helpful in yielding additional information that can contribute to a better understanding of suicide in these minoritized populations. One strategy to potentially address this would be to conduct psychological autopsy interviews with family members and loved ones of IMFs who died by suicide. In addition, the bulk of the qualitative data analysis done in this study was conducted by the study’s Principal Investigator, which may introduce additional bias. However, the themes were checked with other co-authors and experts in the field of occupational health, and the mixed methods study approach helps to triangulate the qualitative findings. Other limitations related to the NVDRS were noted elsewhere. However, a significant number of states began adding data to the NVDRS between 2013 and 2023, and because of this, pooled trends may partially reflect changes in the composition of reporting states versus true changes in suicide patterns. Additionally, some states, notably California, contributed data from only a sample of counties rather than the full state. Therefore, deaths from these states should not be interpreted as representative of the entire state population.

## Conclusion

IMFs are a vulnerable population in the context of the United States workforce, and the intersectional stressors experienced by these workers can lead to suicide. Stigma, substance use, isolation, and social discord intersect with immigration status to create an experience that is untenable for some. Culturally responsive, community-based and workplace interventions are needed to reduce suicide risk and promote mental well-being among immigrant farmworkers.

## Data Availability

The data analyzed in this study is subject to the following licenses/restrictions: data sharing is prohibited without a data use agreement. Requests to access these datasets should be directed to https://www.cdc.gov/nvdrs/about/nvdrs-data-access.html.
